# Commentary on Health service use and costs associated with fluoroquinolone‐related tendon injuries

**DOI:** 10.1002/prp2.821

**Published:** 2021-07-10

**Authors:** Michael Kennedy

**Affiliations:** ^1^ Department of Medicine and Clinical Pharmacology and Toxicology St Vincent’s Hospital University of New South Wales Sydney Australia

Adverse drug reactions will always be a companion to the prescribing of medications. Their occurrence depends on multiple factors including dose of drug, time of administration, genetic factors, age and sex of the patient, and the medical condition for which the medication was prescribed.^1^ A scoping review of 19 studies found reported incidences varying from 6% to 80%, hospitalization rates of 6 to 14%, and mortalities of 0.4% to 2.7%.^2^ Economic evaluation of medication caused adverse drug reactions is challenging because of their wide variety, lack of uniformity in diagnosis, and the need to access necessary medical and other information. A recent systematic review has found that the heterogeneity of costs could be partly explained by methodological differences in the studies.^3^

The present retrospective observational study by Kuula and colleagues examines tendon injuries caused by fluoroquinolones over the period 2002 to 2012. This expands the conclusions from their recent systematic review of health service costs related to fluoroquinolone‐related adverse events.^4^, ^5^ Individual cases are classified as pharmaceutical injuries determined by Finland's Tort Liability Act and relevant guidelines. The Finnish medical system has comprehensive electronic medical system which enables access to inpatient and nonhospital medical records. Claims are logged within each Finnish Hospital District. Compensation costs are borne by the Social Insurance Institution of Finland, municipalities, patients, and the Finnish Pharmaceutical Insurance Pool. The Pharmaceutical Insurance Pool only pays for patients or costs not covered by other parties to the case.

Of 160 claims for fluoroquinolone‐related injury, 146 were considered compensable under the Act, one was excluded as it was not in the Finnish health system, leaving 145 patients eligible for compensation. The 160 reported cases may be less than actually occurs as the authors comment that the compensation system “remains relatively unknown” and there is probably under reporting of tendon injuries.^6^ Of the 145 cases available for review, all patients were ≥18 years of age hence excluding a pediatric population, 99 were male, 46 female. Ages ranged from 21 to 87. Eight claimants died within the timeframe. Diagnoses were classified as tendonitis (52) or ruptures (93).

Most injuries occurred within 3 weeks of commencing fluoroquinolone therapy. Achilles tendons were involved in 97%, 52% received fluoroquinolones for respiratory infections. Of the prescribed fluoroquinolones, 83% received levofloxacin, 9% ciprofloxacin, 5% norfloxacin, ofloxacin, and moxifloxacin 1% each. The prescribed doses are not reported. The increased occurrence of levofloxacin was probably due to the high incidence of pulmonary pathology in the claimants as levofloxacin is often the preferred fluoroquinolone for patients with this condition. Not surprisingly the predisposing factors of age and coadministration of corticosteroids were associated with tendon pathology with odds ratios of 1.03 and 2.25, respectively. About 51% of the patients were hospitalized and the same number of patients were considered to have permanent lesions due to fluoroquinolone use. Surgical treatment was undertaken in 23%. Total direct costs among claimants were 2,146,057 Euros, direct societal costs pre claimant including 14,800 Euros with incapacity and 8744 Euros without incapacity. Fourteen percent (20) of claimants were employed at the time of injury. The insurance pool compensated losses for six claimants aged 39–66 years at an average cost of 50,000 Euros. The average work time lost was 435 h with a final estimate of cost per employed person being 9077 Euros.

Many previous pharmacoeconomic analyses have focused on a single aspect relating to the event such as the extra length of hospitalization, so this wide ranging pharmacoeconomic study is important. It was conducted in a country with a comprehensive system of medical record retrieval from hospitals and outpatient services combined, as well as access to the details of indirect nonmedical societal costs derived from the compensation mechanism. The compensation system in Finland may be unique it its coverage, but similar costs would be occurring wherever similar adverse effects occur. The societal and direct medical costs reported are considerable and as the authors suggest actual costs may well have been higher.

The total costs of adverse drug reactions are rarely considered by prescribing physicians. The present costs may well be different as therapeutic guidelines for the use of fluoroquinolones now recommend a more cautious approach and hence the incidence of fluoroquinolone prescribing is now less than at the time covered in this study.

## DISCLOSURE

The author has no conflicts of interest financial or otherwise.
